# Post‐Cholecystectomy Pancreatitis When Zero Means Lethal: Fulminant Post‐Cholecystectomy Pancreatitis Following a Benign Initial Ranson Score—A Case Report

**DOI:** 10.1002/ccr3.71435

**Published:** 2025-11-04

**Authors:** Fazeela Bibi, Amina Asad, Khalil Elabdi, F. N. U. Mainka, Abdul Quddoos Anwar, Bilal Aslam, Azwa Zubair, Qurat‐ul‐Ain Akram, Fatima Tirmzi, Said Hamid Sadat

**Affiliations:** ^1^ Jinnah Medical and Dental College Karachi Pakistan; ^2^ Nowshera Medical College Nowshera Pakistan; ^3^ Faculty of Medicine and Pharmacy of Rabat Mohammed V University Rabat Morocco; ^4^ Chandka Medical College Larkana Pakistan; ^5^ Nishtar Medical College Multan Pakistan; ^6^ University of Lahore Lahore Pakistan; ^7^ Baqai Medical University Karachi Pakistan; ^8^ Dow University of Health Sciences (DUHS) Karachi Pakistan; ^9^ CMH Lahore Medical College Lahore Pakistan; ^10^ Kabul University of Medical Sciences (KUMS) Kabul Afghanistan

**Keywords:** cholelithiasis, inflammatory response syndrome, multi‐organ failure, open cholecystectomy, post‐cholecystectomy pancreatitis, prognostic scoring, Ranson score, systemic

## Abstract

Post‐cholecystectomy pancreatitis (PCP) represents a rare yet potentially lethal iatrogenic complication whose clinical management is frequently undermined by an insidious progression and the fallibility of traditional prognostic models. We present a compelling case of fulminant PCP in a 46‐year‐old female whose fatal trajectory powerfully illustrates these limitations. Presenting on the fourth postoperative day following an open cholecystectomy, her diagnosis was confirmed by elevated pancreatic enzymes and common bile duct dilation; however, her initial Ranson score was 0, prognosticating minimal risk. After a deceptive 48‐h period of apparent stabilization, her course was marked by a precipitous decline into multi‐organ failure, including acute renal failure, severe hepatocellular injury, and a delayed onset of Systemic Inflammatory Response Syndrome (SIRS) on day seven, culminating in fatal cardiopulmonary arrest. This case demonstrates that a benign initial Ranson score can be a dangerously misleading prognosticator in PCP, establishing the critical need for heightened clinical vigilance to supersede reliance on initial scoring and advocating for a lower threshold for intensive care monitoring irrespective of a seemingly low‐risk presentation.

**Summary**

- Post‐cholecystectomy pancreatitis can present with a deceptively benign Ranson score, masking an impending catastrophic decline. This mandates heightened clinical vigilance and a lower threshold for ICU monitoring, as a low‐risk score is no guarantee of a benign outcome in this specific iatrogenic context.

## 1 Introduction

Acute pancreatitis (AP) is a common gastrointestinal emergency characterized by a wide clinical spectrum [1]. While the majority of cases are mild and self‐limiting, up to 20% can progress to a severe, necrotizing form associated with multi‐organ failure and substantial mortality [2]. Gallstone disease is one of the most prevalent etiologies for AP, and cholecystectomy is the definitive surgical procedure to prevent recurrence [3].

Paradoxically, cholecystectomy itself can precipitate AP. Post‐cholecystectomy pancreatitis (PCP) is an infrequent but potentially lethal iatrogenic complication, with a reported incidence ranging from 0.1% to as high as 15% in some series [4]. Its pathophysiology is multifactorial, with proposed mechanisms including retained microliths, iatrogenic biliary injury, or biliary dysmotility [5]. The clinical trajectory of PCP is not well‐characterized, creating a significant diagnostic and prognostic challenge. Consequently, the utility of traditional prognostic scoring systems like the Ranson criteria—developed and validated for broad etiologies of pancreatitis—is uncertain in this specific post‐operative context [6]. This knowledge gap creates a potential for false reassurance, where a deceptively benign initial score may mask an impending fulminant course and delay the escalation of care.

Herein, we present the case of a 46‐year‐old female who developed fulminant and fatal PCP following an open cholecystectomy. Despite presenting with an initial Ranson score of zero, which typically predicts a low‐risk course, the patient experienced a rapid and irreversible decline into multi‐organ failure and Systemic Inflammatory Response Syndrome (SIRS). This case serves as a critical admonition against over‐reliance on initial severity scoring in the context of PCP. It advocates for a paradigm of heightened clinical vigilance and a lower threshold for definitive pancreatobiliary imaging and intensive care monitoring, irrespective of reassuring initial prognostic indicators.

## 2 History and Examination

### 2.1 Peri‐Operative History

A 46‐year‐old female with a 6‐year history of controlled hypertension underwent an open cholecystectomy for symptomatic chronic cholelithiasis. The decision to perform an open surgery was made after pre‐operative imaging identified severe inflammatory adhesions in the Calot's triangle, which was deemed to pose a high risk for biliary injury with a laparoscopic approach. An intra‐operative cholangiogram was not performed during the procedure. The surgery was reported as otherwise uneventful, and the patient was subsequently discharged on the second postoperative day.

### 2.2 Hospital Admission and Examination

Two days after discharge, which was the fourth postoperative day, the patient presented to the emergency department with a 24‐h history of acute‐onset, severe, and constant epigastric pain that radiated to her right scapula. The pain was exacerbated by meals and was partially relieved when she leaned forward. This was accompanied by nausea, multiple episodes of non‐bilious emesis, and a fever of 38.3°C (101°F). The patient's history was negative for significant alcohol consumption, and a review of her medications revealed no drugs known to be associated with pancreatitis.

Upon physical examination, she was in distress. Her vital signs included a heart rate of 96 bpm, blood pressure of 135/95 mmHg, and an oxygen saturation of 97% on ambient air. Her Glasgow Coma Scale (GCS) score was 13, indicating a state of mild confusion. The abdominal examination revealed tenderness to palpation in the epigastric region and at the surgical site, with an absence of bowel sounds on auscultation. Subtle scleral icterus was also noted.

## 3 Investigations

### 3.1 Initial Diagnostic Workup (Day 0)

Initial laboratory investigations confirmed a diagnosis of acute pancreatitis, with a serum lipase of 268 U/L and serum amylase of 176 U/L. Other significant findings included a leukocytosis of 15.8 × 10^9^/L and an elevated lactate dehydrogenase (LDH) of 320 U/L. An etiologic workup to determine the cause was unrevealing, with a serum triglyceride level of 110 mg/dL (Reference: < 150 mg/dL) and a serum calcium of 9.1 mg/dL (Reference: 8.6–10.3 mg/dL).

An abdominal ultrasound revealed a dilated common bile duct measuring 11 mm in diameter; however, it did not show any visible choledocholithiasis or other obstructing lesions.

### 3.2 Prognostic Scoring and Clinical Status

On admission, prognostic scoring indicated a low risk of severe disease. The Ranson score was 0. The Bedside Index for Severity in Acute Pancreatitis (BISAP) score was 1, which was attributable to her altered mental status (GCS 13). The patient met two of the four criteria for Systemic Inflammatory Response Syndrome (SIRS): a temperature > 38°C and a heart rate > 90 bpm. The Modified Marshall score for organ dysfunction was 0.

Over the initial 48 h, the patient showed signs of stabilization. Her leukocytosis resolved, with the WBC count decreasing to 5.2 × 10^9^/L by Day 2, and her renal function remained normal. The daily SIRS and Modified Marshall scores remained stable at 2 and 0, respectively.

### 3.3 Clinical Deterioration (Day 3–6)

On the third day of admission, laboratory investigations revealed a rapid deterioration. This included a rebound leukocytosis of 16 × 10^9^/L with 88% neutrophilia, the onset of acute renal failure (BUN 168 mg/dL; Creatinine 1.96 mg/dL), and evidence of severe hepatocellular injury, with an AST that rose dramatically to over 10,000 U/L. Consequently, the Modified Marshall score increased to 4, indicating multi‐organ failure involving the renal and hepatic systems.

**FIGURE 1:**
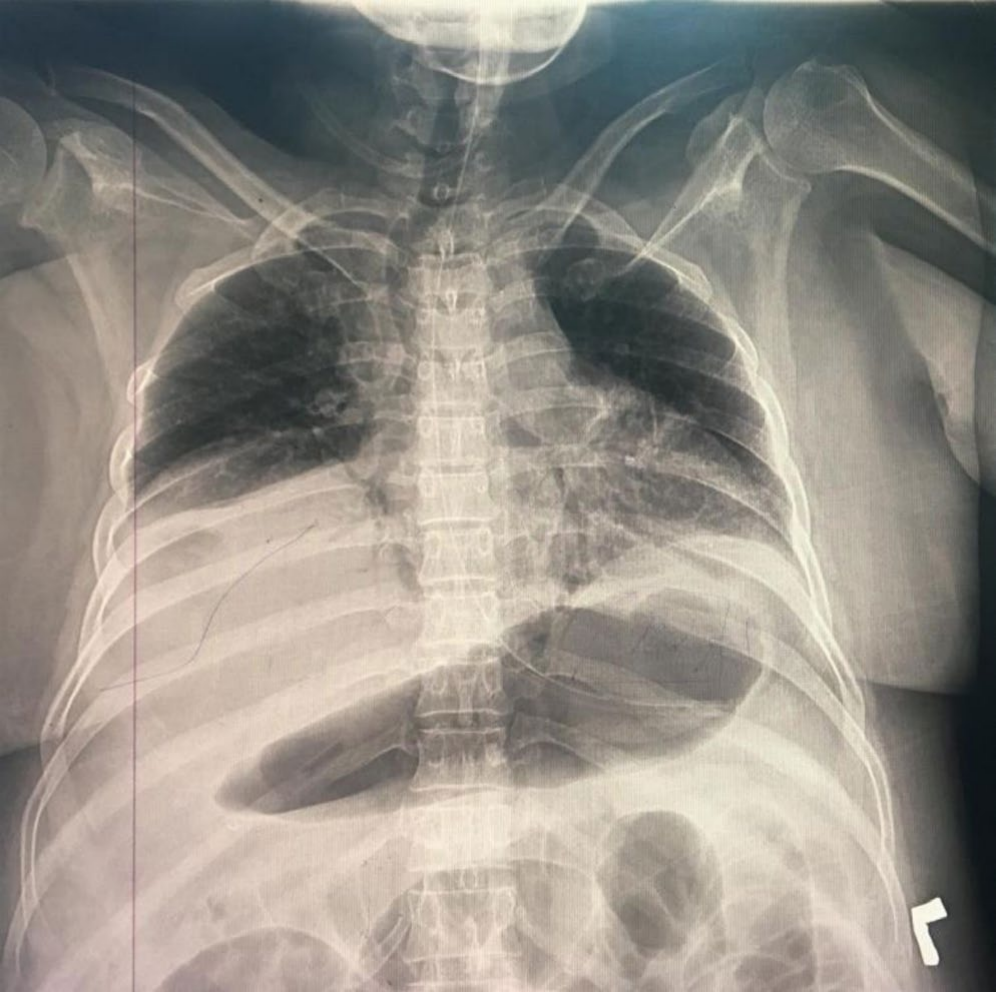
Anteroposterior (AP) supine chest radiograph of the patient on the third day of admission. The image demonstrates a significant left‐sided pleural effusion, a notable pleuropulmonary complication of severe acute pancreatitis. This finding is characterized by the opacification of the lower hemithorax and pronounced blunting of the left costophrenic angle, indicative of substantial fluid accumulation within the pleural space. The presence of such an effusion is consistent with the systemic inflammatory cascade precipitated by the underlying pancreatic pathology.

**FIGURE 2:**
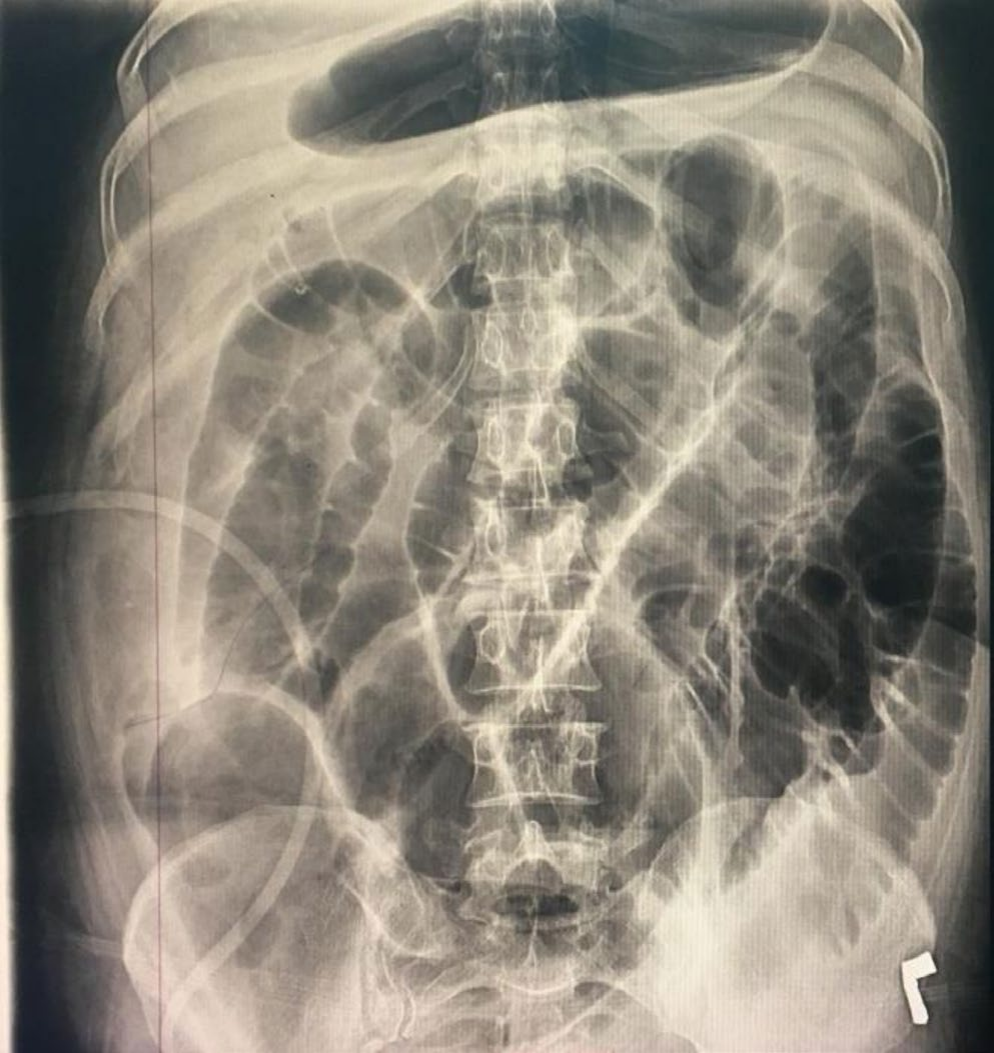
Anteroposterior (AP) abdominal radiograph illustrating the development of paralytic ileus secondary to severe acute pancreatitis. The key radiographic feature is the marked, diffuse dilatation of multiple small and large bowel loops, filled with gas. This generalized intestinal distension, in the absence of a mechanical obstruction, is a classic manifestation of adynamic ileus. This condition arises from the profound inflammatory response and associated peritoneal irritation, leading to a cessation of normal peristalsis and contributing significantly to the patient's clinical deterioration, including absolute constipation.

Her organ dysfunction progressed over the following days. She developed increasing respiratory distress, with her oxygen saturation falling to 91%. A chest radiograph (Figure 1) was performed, which confirmed new bilateral pleural effusions. She also developed absolute constipation, and a subsequent abdominal radiograph (Figure 2) revealed a paralytic ileus. Due to her rapid hemodynamic instability and escalating vasopressor requirements, the patient was deemed too unstable for transport to undergo definitive pancreatobiliary imaging with MRCP or therapeutic intervention with ERCP.

## 4 Treatment and Management

### 4.1 Initial Management (Day 0–2)

The patient was admitted and managed conservatively. Her treatment plan included bowel rest (nil per os), aggressive intravenous fluid resuscitation with Ringer's lactate, and parenteral analgesia with ketorolac. Gastric decompression was initiated with a nasogastric tube. Given the presence of SIRS criteria and the post‐operative context, empiric broad‐spectrum antibiotic therapy was started with intravenous Imipenem/Cilastatin at a dose of 500 mg twice daily.

### 4.2 Escalation of Care (Day 3–7)

Following the patient's deterioration, a therapeutic thoracentesis was performed to address the pleural effusion. The antibiotic regimen was broadened to include amoxicillin‐clavulanic acid.

On the seventh day of admission, the patient fulfilled the criteria for persistent multi‐organ failure and was transferred to the surgical intensive care unit (ICU). Her APACHE II score upon ICU admission was 26, predicting a high mortality rate. Management was intensified with continued aggressive fluid resuscitation and the initiation of dual vasopressor support (norepinephrine and vasopressin) to maintain her mean arterial pressure. Despite receiving high‐flow oxygen via a non‐rebreather mask, her respiratory status continued to decline.

## 5 Outcome and Follow‐Up

The patient's condition proved refractory to maximal medical therapy in the ICU. She suffered a fatal cardiopulmonary arrest on the seventh day of her admission. Blood cultures that had been drawn during her ICU stay were still pending at the time of her death.

## 6 Discussion

This case report details the fatal progression of fulminant post‐cholecystectomy pancreatitis (PCP) in a patient whose initial Ranson score of zero predicted a minimal‐risk course. The profound divergence between this benign prognostic score and the catastrophic clinical reality challenges the reliability of traditional scoring systems in the iatrogenic context of PCP. We argue that this case highlights a distinct clinical phenotype of PCP characterized by a deceptive period of stabilization followed by an irreversible and accelerated decline into multi‐organ failure. This trajectory underscores the fallibility of conventional risk stratification and mandates a paradigm of heightened clinical vigilance in managing this specific post‐operative complication.

Post‐cholecystectomy pancreatitis is a recognized but infrequent complication, with proposed mechanisms including retained microliths, iatrogenic biliary injury, or biliary dysmotility [4]. While its causes are debated, the clinical trajectory and response to standard management protocols are not well‐characterized. Our case is unusual not only in its lethality but in the delayed onset of Systemic Inflammatory Response Syndrome (SIRS) on the seventh day post‐diagnosis. This is a stark contrast to typical presentations of severe acute pancreatitis, where SIRS often manifests within the first 48 h and is a key early predictor of severe outcomes [7]. This atypical timing may suggest a distinct pathophysiological cascade specific to PCP, potentially involving a delayed inflammatory trigger that is not captured by initial prognostic assessments.

The failure of the Ranson score in this context is a key finding. The Ranson criteria, while still considered a valid tool in many settings [8], were validated primarily in pancreatitis of alcoholic or biliary etiology and may not adequately capture the inflammatory drivers of iatrogenic, post‐operative pancreatitis. The initial 48‐h period of apparent stabilization, with normalizing leukocyte counts and a static Ranson score of 0, may represent a particularly insidious feature of this condition. This “lucid interval” could have been followed by a “second hit”—perhaps from a complete obstruction by a retained microlith or a post‐operative ischemic event—triggering an explosive and overwhelming inflammatory cascade that was not reflected in the initial admission parameters. While some scoring systems like APACHE II may offer better predictive accuracy for severity and mortality [8], the fundamental lesson from this case is that reliance on any initial score can foster a dangerously false sense of security.

A critical aspect of this case was the precipitous onset of multi‐organ failure, including acute renal failure and severe hepatocellular injury, with AST levels exceeding 10,000 U/L. This degree of liver injury is atypical for pancreatitis alone and is more suggestive of ischemic hepatitis (“shock liver”) secondary to the patient's fulminant septic shock and profound hemodynamic instability. This rapid deterioration directly impacted management. While definitive pancreatobiliary imaging with MRCP or therapeutic ERCP is indicated for suspected biliary obstruction, the patient's rapid hemodynamic collapse and prohibitive vasopressor requirements rendered her too unstable for transport or intervention. Her clinical trajectory tragically illustrates a scenario where the disease's virulence outpaced the window for safe and effective source control intervention.

We acknowledge the inherent limitations of a single case report. The exact etiology, such as a retained microlith, was not definitively confirmed as an autopsy was not performed, and blood cultures were pending at the time of death. Therefore, while our observations are compelling, they cannot be generalized. However, the report serves as a powerful, hypothesis‐generating account that warrants further investigation into the unique pathophysiology of PCP.

In conclusion, this case of fatal post‐cholecystectomy pancreatitis demonstrates that a benign initial Ranson score can be dangerously misleading in this specific clinical setting. The key clinical lessons are threefold: First, PCP can present with a deceptively stable period before catastrophic decline, mandating heightened clinical vigilance irrespective of initial scores. Second, a lower threshold for transfer to an intensive care setting should be considered for PCP patients, even those appearing to be “low‐risk”. Finally, this case underscores a critical research gap and highlights the urgent need for multicenter registries to better define the natural history of PCP and develop more sensitive, context‐specific prognostic tools, potentially incorporating dynamic biomarkers like IL‐6 or procalcitonin.

## 7 Conclusion

The fatal trajectory of this case of fulminant post‐cholecystectomy pancreatitis serves as a stark admonition against over‐reliance on traditional prognostic models in this iatrogenic setting. It demonstrates that a deceptively benign Ranson score can mask an impending catastrophic decline, thereby invalidating a wait‐and‐see approach. The primary clinical lesson is that for patients with PCP, unwavering clinical vigilance must supersede initial risk stratification scores. We advocate for a paradigm shift toward a lower threshold for escalation of care, including early consideration for intensive care monitoring, even in patients who appear clinically stable. Ultimately, this case underscores a critical research gap and the urgent need to develop more sensitive prognostic tools specific to PCP, as a benign score is no guarantee of a benign outcome.

## Author Contributions

**Fazeela Bibi:** conceptualization, data curation, formal analysis, investigation, methodology, project administration, validation, visualization. **Amina Asad:** conceptualization, data curation, formal analysis, investigation, methodology, project administration. **Khalil Elabdi:** conceptualization, data curation, formal analysis, investigation, methodology, project administration, resources, validation, writing – original draft, writing – review and editing. **F. N. U. Mainka:** conceptualization, formal analysis, methodology. **Abdul Quddoos Anwar:** data curation, formal analysis, investigation, methodology, resources. **Bilal Aslam:** conceptualization, data curation, investigation, methodology, project administration. **Azwa Zubair:** data curation, formal analysis, investigation, methodology. **Qurat‐ul‐Ain Akram:** conceptualization, data curation, formal analysis, investigation, methodology, project administration. **Fatima Tirmzi:** investigation, methodology, project administration, validation, visualization. **Said Hamid Sadat:** validation, writing – original draft, writing – review and editing.

## Ethics Statement

The authors have nothing to report.

## Consent

Written consent was taken from the patient's father. The patient family is contact with authors.

## Conflicts of Interest

The authors declare no conflicts of interest.

## Data Availability

The data was taken from a patient who presented to our hospital, all data and references are publicly available on databases such as Pub‐med and Google Scholar.
